# Surgical management of sickle cell retinopathy: a systematic review of clinical outcomes and a single-arm meta-analysis of single-surgery anatomical success

**DOI:** 10.3389/fopht.2026.1842211

**Published:** 2026-07-23

**Authors:** Maher Nassor, Ahmed Bakr, Usman Muhammad, Marwan Tahoun

**Affiliations:** 1 School of Medicine, University of Sunderland, Sunderland, United Kingdom; 2 School of Medicine, University of Liverpool, Liverpool, United Kingdom; 3 School of Medicine, University of Manchester, Manchester, United Kingdom

**Keywords:** pars plan vitrectomy, sickle cell disease, sickle cell retinopathy, surgical outcomes, vitreoretinal surgery

## Abstract

**Introduction:**

Sickle cell retinopathy is a consequence of sickle cell disease that results from vascular complications. Sickle cell disease is predominantly prevalent in Africa, evidenced by 20% to 30% of the population in Gabon, Cameroon, and Ghana being affected by it. This systematic review aims to evaluate the surgical outcomes of patients with sickle cell retinopathy to inform evidence-based management of late stages of sickle cell retinopathy.

**Methods:**

This systematic review was conducted in accordance with the PRISMA 2020 guidelines. A Literature search was conducted on PubMed, Embase (Ovid), and Cochrane (CENTRAL) on 05/08/2025. Inclusion criteria included studies with patients with sickle cell retinopathy who underwent surgery and reported anatomical and/or visual outcomes. A random effects single-arm meta-analysis was carried out in the anatomical success group using JASP software, and the risk of bias was evaluated using JBI and RoB2 tools.

**Results:**

7 studies, comprising 408 eyes, were included. Overall, the evidence showed that surgical treatment in patients with sickle cell retinopathy results in improved final visual acuity and high single-surgery anatomical success (SSAS, between 60% to 83%). The pooled SSAS data, based on logit-transformed proportions, yielded an effect of 0.83 (95% CI: 0.24–1.42), corresponding to a pooled SSAS of approximately 70% (95% CI: 56–80%), with low, non-significant heterogeneity among the included studies (Q (4) = 5.61, p = 0.230; I² = 29.73% [0.00, 92.46]). Leave-one-out sensitivity analysis confirmed that the estimate was robust, with the pooled SSAS ranging from approximately 66% to 74% across iterations.

**Discussion:**

Studies have reported a considerable gain in final visual acuity and anatomical success in patients undergoing surgery for sickle cell retinopathy. The small number of studies and small sample sizes increase heterogeneity. Therefore, although the results indicate favourable surgical outcomes, clinicians must weigh up the benefits of surgery against possible complications. In conclusion, more studies are warranted to guide evidence-based medicine.

**Systematic review registration:**

https://www.crd.york.ac.uk/PROSPERO/view/CRD420251165357, identifier CRD420251165357.

## Introduction

Sickle cell disease (SCD) is an inherited hemoglobinopathy caused by a mutation in the β-globin gene, leading to inflammation, recurrent vaso-occlusion, chronic haemolysis, and ischaemic organ damage (1). It mainly affects people of African descent, as data shows that between 20 to 30% of individuals carry the sickle cell disease in countries like Gabon, Ghana, and Cameroon. Globally, around 5% of people carry the gene for haemoglobin disorders, which include sickle cell disease and thalassaemia (2). This gives SCD a substantial prevalence both nationally and globally.

Sickle cell retinopathy (SCR) is a retinal vascular complication of SCD that can greatly impair visual acuity. This develops due to the high sensitivity of the retina to ischaemic damage. SCR can be divided into proliferative SCR (PSCR) and non-proliferative SCR (NPSCR) forms. NPSCR is characterised by “salmon patch” haemorrhages, ischaemic retinal changes, and peripheral arteriolar occlusions (1). Contrastingly, PSCR is characterised by peripheral neovascularisation that can result in retinal detachment (RD) and vitreous haemorrhage (VH), leading to severe visual impairment (3). Consequently, PSCR is a more sight-threatening form of SCR.

Several genotypes of SCD with differing ocular risk and clinical features present with different incidence rates of PSCR. For instance, the homozygous SS disease (most severe systemic form of sickle cell disease) has a paradoxically very low incidence rate of PSCR (3%). The most important genotypes, which usually present significant ophthalmic manifestations, include Sthal disease (HbSβ-thalassemia) and heterozygous SC (HbSC), accounting for 14% and 33% incidence of PSCR, respectively (4). This is supported by a long-term Jamaican cohort study found that by around 25 years, PSCR had developed in 43% of HbSC patients and in 14% of HbSS patients (5). Due to this high risk of PSCR development, regular ophthalmologic screening is recommended, starting around the age of 10 with periodic dilated fundoscopy (1). Early stages of PSCR lesions may regress or be treated with peripheral scatter laser photocoagulation, but late stages often require surgery. In Severe PSCR, persistent VH (non–clearing) or RD necessitates Pars Plana Vitrectomy (PPV, sometimes combined with scleral buckling and long–acting tamponade) for blood clearance and retina reattachment (5).

Given the potential for vision loss from advanced PSCR, good insights of surgical outcomes are essential. Nevertheless, high-quality surgical data is limited. Therefore, this systematic review aims to collate published data of vitreoretinal surgery, summarising anatomical success rates, visual outcomes, and pathologies associated with PSCR to inform evidence-based management of patients with advanced PSCR.

## Methods

### Registration

This review is reported in accordance with the Preferred Reporting Items for Systematic Reviews and Meta-Analysis (PRISMA) 2020 guidelines (6) and prospectively registered on Prospero (CRD420251165357).

### Eligibility criteria

The inclusion criteria were patients with any genotype of sickle cell disease and a diagnosis of PSCR, any ocular surgical intervention, and outcomes such as visual acuity, retinal attachment, regression of neovascularisation, and complications. Single-surgery anatomical success (SSAS) is defined as the proportion of patients whose retina is attached after one surgery without requiring an additional retinal reattachment surgery (7). Study designs eligible for inclusion were randomised controlled trials (RCTs), cohort studies, case-control studies, and case series. Exclusion criteria included studies not reporting surgical outcomes separately, case reports, review articles, editorials, and conference abstracts without full text.

### Information sources

The following databases were searched on 05/08/2025 (with no date restriction): PubMed, Embase via Ovid, and Cochrane (CENTRAL). The reference list of included studies was exported from the relevant databases and imported into Rayyan for screening (8).

### Search strategy

A database-specific search strategy was developed with a combined controlled vocabulary (E.g., MeSH Terms). Full search strategies for each database are provided in the **Supplementary Material**. No language restrictions were applied. Results were exported to Rayyan for screening (8). In PubMed, a combination of MeSH terms and free-text terms was used to search for sickle cell disease (“sickle cell disease”, “sickle cell anaemia”, “sickle cell”), retinopathy (“retinopathy”, “sickle cell retinopathy”), and surgical interventions (“surgery”, “surgical procedures”, “vitrectomy”, “photocoagulation”, “anti-VEGF”, “laser”). Similar search strategies were implemented in Embase using Emtree controlled vocabulary and free-text terms, and in Cochrane CENTRAL using keyword searches.

### Study selection

Titles and abstracts were screened independently by two reviewers (MN and AB) using Rayyan (7). Articles deemed potentially eligible for inclusion underwent full-text screening by the same two reviewers (MN and AB), who screened independently. Disagreements at any stage were resolved by discussion between the same two reviewers. We recorded reasons for exclusion at full-text screening and present the study selection process using the PRISMA 2020 flow diagram (9).

### Data extraction

A piloted data extraction form was utilised. A single reviewer (MN) independently extracted the data, and the data were peer-reviewed by the supervisor (MT). Extracted items included study identifiers (author, country, year), study design, setting, sample size, patient demographics, preoperative retinal findings, retinal detachment characteristics, types of surgical interventions, Anatomical and Visual outcomes (primary outcomes), and complications (secondary outcomes).

### Risk of bias assessment

Risk of bias was assessed independently by a single reviewer and peer reviewed by the supervisor. For retrospective case series and clinical trials, we used the Joanna Briggs Institute (JBI) Critical Appraisal Tool and the Revised Cochrane Risk of Bias (RoB) tool for randomised trials (RoB2, 10, 11). Traffic light plots were generated using the RobVis tool (12). A summary of risk of bias tables and domain-level judgement is provided in the **Supplementary Material**.

### Data synthesis

Following full-text screening, the characteristics and outcomes of studies were compared against the predefined inclusion criteria. Studies reporting surgical interventions and outcome measures were analysed and grouped into the following: SSAS, perioperative visual acuity (VA), final VA, and complications.

### Meta analysis

Where enough data were available, a quantitative synthesis was conducted through meta-analysis focusing on SSAS. A random effects meta-analysis of single proportions was conducted using JASP statistical software (Version JASP 0.95.2, 13). Studies that did not explicitly report SSAS outcomes were excluded from the meta-analysis. Pooled incidence estimates are presented with 95% confidence intervals (CIs), and prediction intervals (PIs) were calculated to reflect the anticipated range of effects in future studies.

Statistical heterogeneity was assessed using Cochran’s Q statistic, with heterogeneity was evaluated with the I^2^ statistic. I^2^ values of approximately 0% to 40%, 30% to 60%, 50% to 90%, and 75% to 100% were interpreted as representing no heterogeneity, low heterogeneity, moderate heterogeneity, and considerable heterogeneity, respectively (14).

Sensitivity analyses were conducted by excluding one study at a time. All pooled estimates for SSAS were interpreted descriptively as no direct comparative meta-analysis was possible due to the limiting nature of the included studies. All statistical analyses were performed using where statistical significance was set at p < 0.05.

## Results

**Figure 1:**
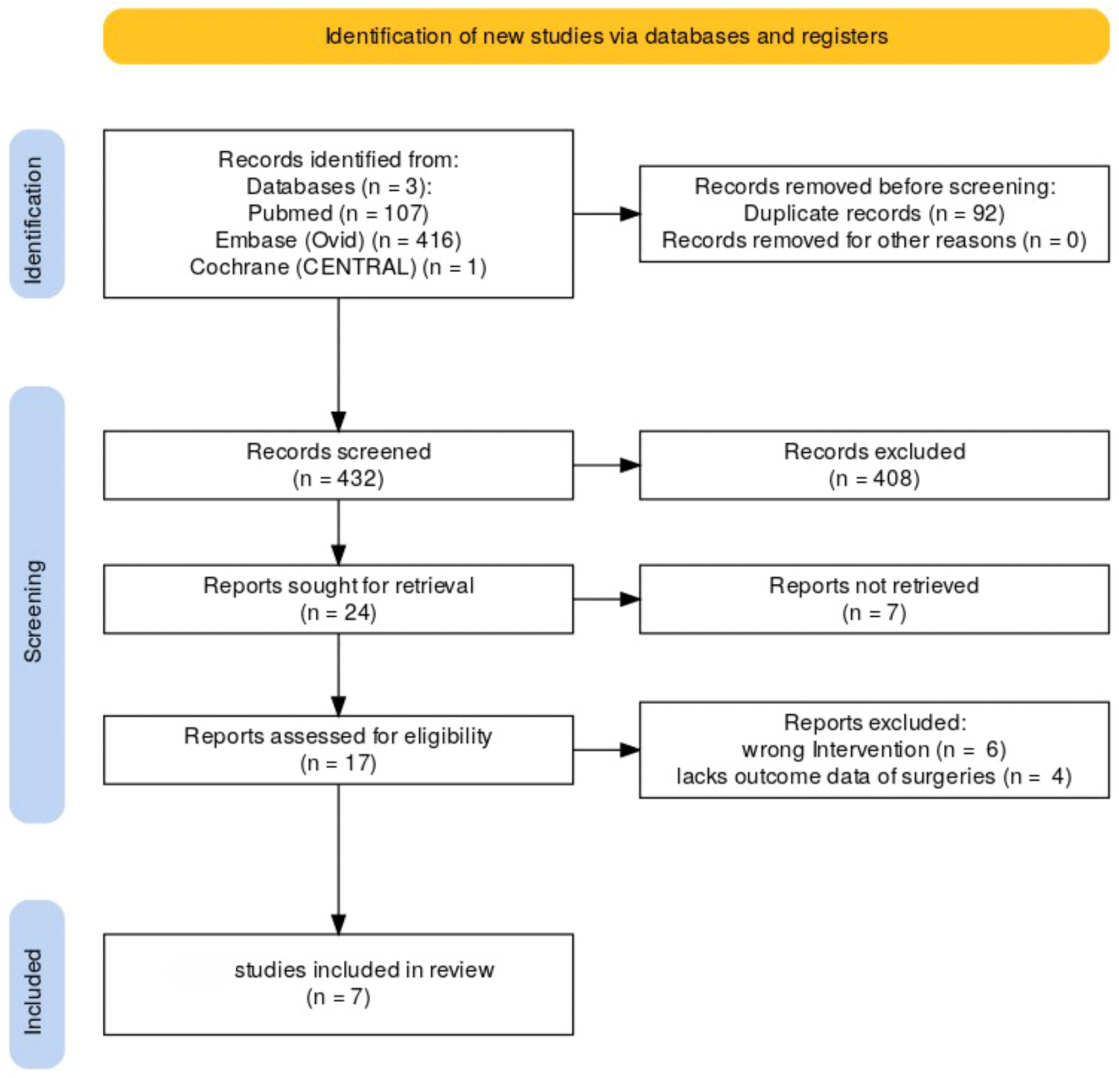
PRISMA flow diagram of study selection showing the process of identification, deduplication, title-and-abstract screening, and full text screening.

A comprehensive literature search conducted on the three databases yielded articles. After the removal of 92 duplicates, 432 articles were screened for abstract and title; 18 records underwent full-text review. Following quality assessment of full text screening, 7 studies were included in this review, as shown in **Figure 1**.

### Risk of bias

**Figure 2:**
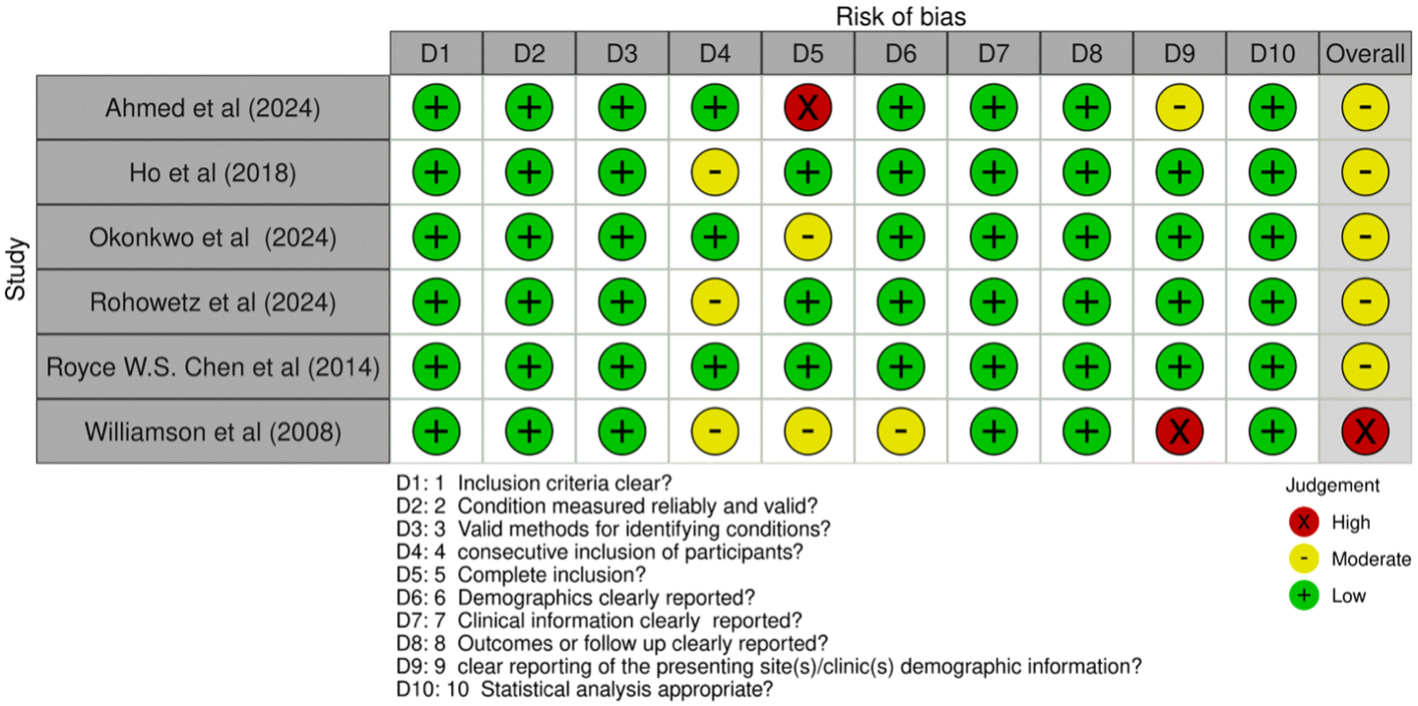
A risk of bias assessment for the included case series. Traffic light plots display the individual domains and the overall risk-of-bias assessments for each case series (Yes = green, No = red, unclear = grey).

**Figure 3:**
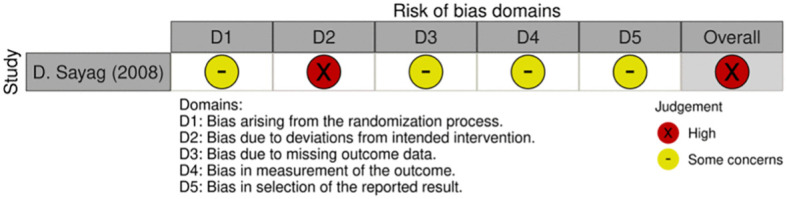
Risk of bias assessment for the included prospective clinical trial. Most domains were rated “some concerns” (yellow), indicating moderate risk. The overall risk of bias was rated high.

The risk of bias was assessed using the JBI critical appraisal checklist for case series and Rob2 for the prospective clinical trial. **Figures 2** and **3** present traffic plots generated with the RobVis tool, which summarise judgments across domains for the included studies and their overall risk of bias. Most case series exhibited an overall moderate (yellow) risk, and the prospective clinical trial was assessed to have a “high” risk of bias. 

### Anatomical outcomes (SSAS)

Across the included studies reporting SSAS, the rate of SSAS ranged from approximately 60% to 83.3% (Chen et al. 60%; Rohowetz et al. 61.5%; Ahmed et al. 70%; Ho et al. 78.9%; Williamson et al. 83.3%, 15–17, 20, 21). Okonkwo et al. (n=108) and Sayag et al. (n=101) did not report SSAS, which limits comparison within the largest cohorts. Overall, SSAS was moderate, with failures mostly attributed to various complications, including proliferative vitreoretinopathy (PVR) or RD, where documented (15).

### Visual outcomes

Most studies indicated improvements in postoperative visual acuity. However, reporting methods varied, including differences in Snellen ranges, macular status subgroups, and logMAR means (**Table 1**). For studies reporting mean logMAR values, visual acuity improved from approximately 1.07 to 0.42 logMAR (Williamson et al; roughly 20/200 to 20/50) and from 1.30 to 0.74 logMAR (Ho et al; approximately 20/400 to 20/110, 20, 21). Okonkwo et al. documented improvement from 1.77 logMAR to around 0.55–0.58 logMAR, depending on the adjuvant strategy employed (18). Rohowetz et al. presented outcomes stratified by macular status, demonstrating better final visual acuity in eyes with preserved maculae than in those with macular involvement, highlighting macular status as a significant factor affecting outcomes (16) (**Table 2**).

**Table 1:** Summary of study and patient characteristics, surgical type, outcomes, and setting of the included studies.

First author (year)	Study design	Study population (participants, demographics)	Intervention (surgical details)	Outcomes measured	Setting
(15) Ishrat Ahmed (2024)	Retrospective case series	28 patients (30 eyes); Mean Age: 42.1 ± 15.1 yrs; Sex: 16F, 12M; Genotype: HbSC (72.2%), HbSS (16.7%), Trait (11.1%); Disease: Proliferative SCR	PPV (83.3%) or PPV + Scleral Buckle (16.7%); Membrane peel & endolaser in all eyes; Tamponade: Silicone Oil (54.8%), Gas (29.0%), Air (9.7%)	SSAS; Final Reattachment; Recurrent RD; Time to Recurrence; Cause of Recurrence; Pre-op and Final VA; VA Change (≥3 lines); Complications	Tertiary Care Centre
(16) Landon J Rohowetz (2024)	Retrospective case series	61 patients (65 eyes); Mean Age: 41 years; Genotype: HbSC (44.4%), HbSS (25.9%), Trait (24.1%), Sβ-thal (5.6%); Disease: Proliferative SCR	PPV (55.4%) or PPV + Scleral Buckle (44.6%); Endolaser in all cases; Gauge: 23G (55.4%), 25G (41.5%)	SSAS; Pre-op and Final VA (by subgroup); Complications	Tertiary Care Centre
(17) Royce W S Chen (2014)	Retrospective case series	14 patients (15 eyes); Mean Age: 48 yrs; Sex: 6F, 8M; Genotype: HbSC (9 patients), Sβ-thal (1 patient), HbAS Trait (4 patients); Disease: Proliferative SCR	PPV (100%); PPV + Scleral Buckle (13.3%); Membrane peel; Tamponade: Silicone Oil, C3F8, SF6, Air	SSAS; Recurrent RD; Pre-op and Final VA; Reoperations; Complications	Tertiary Care Centre
(18) Ogugua Ndubuisi Okonkwo (2024)	Retrospective case series	88 patients (108 eyes); Mean Age: 38.9 yrs; Sex: 40F, 48M; Genotype: HbSC (76.9%), HbSS (17.6%), Trait (5.5%); Disease: Proliferative SCR	PPV + Scatter Laser (29 eyes); PPV + IVI Anti-VEGF + Scatter Laser (20 eyes)	Pre-op and Final VA; VA Change (Snellen lines)	Tertiary Care Centre
(19) D. Sayag (2008)	Prospective clinical trial	101 patients (101 eyes); Mean Age: 29 yrs; Sex: 62F, 39M; Genotype: HbSS (52.1%), HbSC (47.9%); Disease: Proliferative SCR (Graded A-E)	Intervention for complications: PPV (for VH), Scleral Buckle (for RD)	VA Stability/Change; Rate of RD and VH in untreated eyes; Anatomical and Visual Outcome in surgically treated eyes	Clinical Trial Setting
(20) Williamson, TH (2009)	Retrospective Case Series	27 patients (18 eyes); Mean Age: 41 yrs; Sex: 21F, 6M; Genotype: HbSC (25 eyes), HbSS (2 eyes); Disease: Proliferative SCR	PPV (100%); Membrane peeling; No scleral buckles; Tamponade: SF6, C3F8, Air, Silicone Oil	SSAS; Final Anatomical Success; Recurrent RD; Pre-op and Final VA; VA Improvement; Complications	Tertiary Care Centre
(21) Ho, J (2018)	Retrospective case series	63 patients (71 eyes); Mean Age: 41.7 yrs; Sex: 29F, 34M; Genotype: HbSC (87.3%), HbSS (9.9%), Other (2.8%); Disease: Proliferative SCR	PPV (100%); Retinectomy, ILM peel, Laser; No scleral buckles; Tamponade: Silicone Oil (28.2%), C3F8, SF6	SSAS; Final Attachment; Recurrent RD; Pre-op and Final VA; VA Change; Reoperations; Complications	Tertiary Care Centre

PPV, Pars Plana Vitrectomy; VA, Visual acuity; Pre-op, preoperative visual acuity; Post-op, Postoperative visual acuity; G, Gauge; VH, Vitreous haemorrhage; RD, Retinal detachment. HbSC, Haemoglobin Sc disease; HbSS, Haemoglobin SS; Sβ-thal, Sickle Beta-Thalassemia; ILM, Internal limiting membrane; IVI, intravitreal injection; F, Female; M, Male.

**Table 2:** Summary of surgical outcomes Illustrating SSAS, VA, and complications of patients with sickle cell retinopathy.

Author (year)	Eyes (n)	SSAS (%)	Preoperative VA	Final VA	Complications
(15) Ahmed et al. (2024)	30	70	20/600	20/100	Recurrent RD (43%, mainly PVR); Epiretinal membranes (50%); cataract (39%)
(16) Rohowetz et al. (2024)	65	61.5	~20/800 (macula-involving RD); ~20/300 (macula-sparing RD)	~20/80 (macula-sparing RD), ~20/500 (macula-involving RD)	Ocular hypertension (11.1% PPV, 13.1% PPV + SB); neovascular glaucoma (1 eye); iris neovascularisation (1 eye); hyphema and anterior inflammation (1 eye); progressive optic disc cupping (1 eye)
(17) Chen et al. (2014)	15	60	20/200-LP	20/20-20/400	Cataract progression (7 eyes); ERM (1 eye); hyphema and IOP rise (1 eye)
(18) Okonkwo et al. (2024)	108	NR	1.77 logMAR	0.58 logMAR (vitrectomy + SRLP); 0.55 logMAR (Vitrectomy + IVI + SRLP)	NR
(19) Sayag et al. (2008)	101	NR	Mostly ≤20/200	20/20-20/50 (SSAS eyes)	Untreated patients with elevated sea fan; complete fibrosed sea fan; haemorrhage
(20) Williamson et al. (2009)	18	83.3	1.07 logMAR (~20/200)	Mean 0.42 logMAR (~20/50)	Iatrogenic breaks (38.9%); cataract (16.75%); post-op vitreous cavity haemorrhage (11.1%); post-op uveitis (1 eye)
(21) Ho et al. (2018)	71	78.9 (30/38 RD eyes)	Mean 1.30 logMAR (~20/400)	Mean 0.74 logMAR (~20/110)	Giant retinal tears (2.8%); intra-op iatrogenic breaks (8.5%); post-op fibrinous uveitis (1 eye); branch retinal artery occlusion (1 eye); hypertensive uveitis (1 eye)

NR, not reported; PPV, pars plana vitrectomy; SB, scleral buckle; VA, visual acuity; PVR, proliferative vitreoretinopathy; ERM, epiretinal membrane; IOP, intraocular pressure; SRLP, Scatter Retinal Laser Photocoagulation; IVI, intravitreal injection.

### Meta-analysis of SSAS

**Figure 4:**
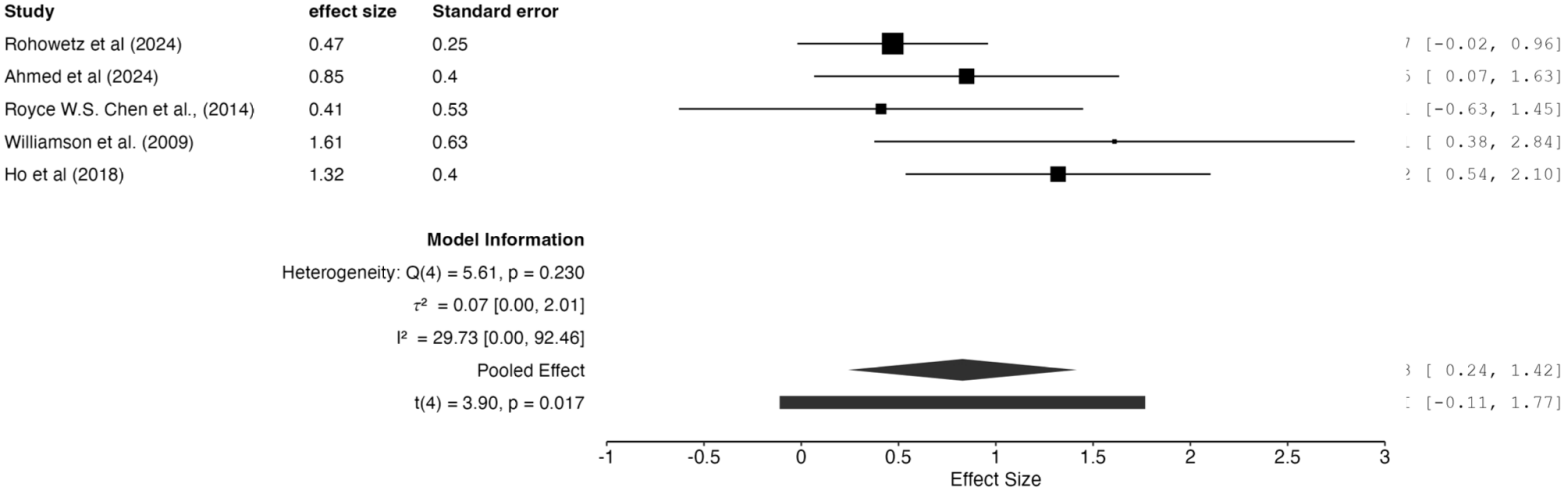
Forest plot illustrating pooled SSAS in patients who underwent surgery for sickle cell retinopathy.

A random-effects meta-analysis was performed to assess the anatomical success rate following a single surgical procedure in patients with sickle cell retinopathy. Five studies were included, covering data from 2009 to 2024. The pooled effect size was 0.83 (95% CI: 0.24–1.42); t (4) = 3.90, p = 0.017), indicating a statistically significant overall success rate following one surgical intervention. Heterogeneity among the included studies was low and non-significant (Q (4) = 5.61, p = 0.230; I² = 29.73% [0.00, 92.46]), suggesting that variability in effect sizes was largely consistent with chance (**Figure 4**).

The between-study variance was estimated at τ² = 0.07 [0.00, 2.01]. Effect sizes across individual studies ranged from 0.41 (Chen et al) to 1.61 (Williamson et al, 17, 20). Although most studies showed positive effect estimates, the confidence intervals varied considerably, reflecting differences in sample sizes and study precision. Overall, these results support a favourable anatomical success rate following a single procedure for sickle cell retinopathy, though the small number of studies warrants caution in interpretation.

### Sensitivity analysis

A leave-one-out sensitivity analysis was performed to evaluate the robustness of the pooled estimate and to determine if any single study disproportionately influenced the results. Influence of Individual Studies: Removing any one study did not significantly change the overall direction of the pooled effect, which remained positive across all analyses. Heterogeneity remained low to moderate throughout, and statistical significance was retained in every analysis except after the exclusion of Ahmed et al.

The removal of Ho et al. had a notable effect. The model heterogeneity became non-significant (Q(3) = 3.30, p = 0.348), and the I² statistic decreased to nearly 0% (0.004%). The pooled effect stayed significant but was greatly reduced in size to 0.65 (95% CI: 0.02 to 1.27, p = 0.046). This indicates that Ho et al. accounted for most of the modest heterogeneity observed in the primary analysis and slightly elevated the pooled effect. S3 File.

Excluding Rohowetz et al. produced a slightly larger pooled effect (1.03, p = 0.021) with no residual heterogeneity (I² ≈ 0%), whereas excluding Williamson et al. produced a slightly smaller effect (0.73, p = 0.036) with low heterogeneity (I² ≈ 24%); both remained statistically significant. S4 and S5 Files.

Removing Ahmed et al. rendered the pooled effect marginally non-significant (0.86, p = 0.056), whereas removing Chen et al. left it significant (0.92, p = 0.033); both point estimates remained substantial, with moderate heterogeneity (I² ≈ 46% and 42%, respectively). S1 and S2 Files.

## Discussion

The collective evidence from the included studies suggests that current vitreoretinal surgery can yield high anatomical success and improved vision in PSCR. For example, Ahmed et al. reported a final reattachment rate of 93.3% in eyes with 70% SSAS (15). Similarly, Williamson et al. and Ho et al. reported SSAS of 83.3% and 78.9%, respectively, after vitrectomy (20, 21). Eyes treated for epiretinal membrane (ERM) or vitreous haemorrhage alone achieved better postoperative vision, with 20/20 to 20/60 vision (17). However, eyes treated with mixed tractional/rhegmatogenous detachments had poorer results (15). Achieving complete retinal reattachment appeared important across most studies because eyes with recurrent or residual detachment generally had poor visual outcomes (15, 17).

Some studies suggest that combined interventions may improve SSAS. This is observed in Rohowetz et al., who reported a higher single–operation success rate in eyes managed with combined scleral buckle (SB) and vitrectomy (72.4%) than with vitrectomy alone (47.8%, 16). Complementing this, Ahmed et al. identified long-term tamponade use as a predictor of lower single-surgery success, highlighting the multifactorial nature of anatomical stability (15). Consistent with these results, Okonkwo et al. also reported that combined interventions (vitrectomy with laser photocoagulation) yielded the largest VA gains (+3.07 and +2.10 Snellen lines without and with anti-vascular endothelial growth factor (anti-VEGF), respectively), compared with SRLP alone (18). These findings suggest that careful use of adjuncts during surgical procedures in patients with retinopathy may help increase SSAS.

VA outcomes showed consistent patterns when stratified by presentation. Eyes with Macula-off detachments generally had poorer vision than macula-on or only VH. For instance, in Rohowetz et al., Macula sparing RD improved from 20/298 to 20/77 and isolated VH eyes from 20/944 to 20/56, while macula-involving RD averaged ~20/500 at final follow-up (16). Comparably, Chen et al. found that all eyes treated for VH or ERM improved in vision, but 4 of the 8 eyes treated for tractional/rhegmatogenous detachments developed recurrence and had a moderate improvement in vision (20/100 – 20/400, 17). Williamson et al. also noted a mean improvement from a mean logMAR 1.07 (Snellen ∼6/70) pre-op to 0.42 (Snellen∼6/15) post-op (20). In Ho et al., the overall mean change corresponded to a gain of roughly 5.5 Early Treatment Diabetic Retinopathy Study (ETDRS) lines (21). In summary, visual rehabilitation appeared evident in most cases across studies when anatomical success was achieved. Overall, studies suggest that successful reattachment may be accompanied by gains in VA.

The meta-analysis indicates a generally favourable rate of single-surgery anatomical success (SSAS) following intervention for sickle cell retinopathy, with a pooled SSAS of approximately 70% (95% CI around 56-80%) derived from back-transformed logit estimates in JASP. Heterogeneity was low and non-significant (I² ≈ 30%; Q(4) = 5.61, p = 0.23), indicating relatively consistent effect estimates across studies, although some residual variation may reflect differences in case mix, such as macular involvement or proliferative vitreoretinopathy, surgical techniques, follow-up durations, and SSAS definitions. Leave-one-out sensitivity analysis confirmed that the pooled estimate was robust to the removal of any single study: the pooled SSAS ranged from approximately 66% (omitting Ho et al.) to 74% (omitting Rohowetz et al.), with a consistent effect direction and low heterogeneity (I² ≤ 46%) across all iterations. The pooled effect remained statistically significant in every iteration except after the removal of Ahmed et al., where it became marginal (p = 0.056). Overall, surgical intervention appears effective for achieving anatomical success in many patients; however, standardised, stratified prospective reporting is necessary to enhance the precision of estimates and clarify sources of between-study variability.

## Complications

Almost all case series included emphasised that complications are common in PSCR surgery. Although most studies reported a substantial VA improvement and moderate to high SSAS ranging from 60% (17) to 83% (20), recurrent RD and PVR remain an important cause of failure in retinal surgical procedures. A consistent cause of recurrent RD cases has been observed to be PVR in several studies, despite meaningful SSAS. This is shown in Ahmed et al. (a large multicentre retrospective case series), who reported SSAS of 70% and recurrent RD in 43% of eyes, of which 76.9% were driven by PVR (15), highlighting PVR as the predominant cause of retinal detachment. This is consistent with Chen et al., who reported SSAS of 60%, but PVR in 100% of the recurrent RD cases (17). Other complications include intraoperative iatrogenic breaks during vitrectomy (38.9% and 8.5% of the cases in Williamson et al. and Ho et al, respectively, 20, 21). Importantly, no study was found to report a high rate of anterior segment ischaemia after surgery. Nonetheless, the challenges PSCR surgery faces, even with skilled surgeons, additional procedures are often needed (E.g. more than 15% of patients required secondary retinal reoperations in Ho et al. (21)).

## Limitations

The existing evidence is limited. All studies except Sayag et al. (a prospective clinical trial) are retrospective case series. There is no randomised clinical trial that compares surgical vs non-surgical management of proliferative SCR or compares different surgical techniques. Sample sizes are typically small (vary between 15 and 108 eyes (17, 18)), and treatment protocols are inconsistent. For example, Okonkwo et al. utilised multiple modalities (anti-VEGF, vitrectomy, and laser) without randomised assignment (18). In addition, most studies were rated moderate to high risk of bias because of the retrospective design, Unclear patient selection strategy, and insufficient reporting. The heterogeneity that exists complicates direct comparison of outcomes.

Variation is also present in the outcome definitions. For example, such as single–surgery success versus any reattachment and final VA at a fixed timepoint versus at last follow-up. In addition, publication bias was not assessed because the number of included studies was fewer than 10. These issues may introduce risk of bias and limit the precision of pooled estimates. Publication bias is possible because successful surgical outcomes may be more reported than failures. Therefore, the reported success rates (E.g. 60 – 83% SSAS) should be interpreted cautiously. The only prospective trial included in this systematic review did not deal with advanced detachments but focused mainly on the photocoagulation of early neovascular sea fan lesions (19). It is important to note that no clear benefit of scatter laser in mild “sea fan” lesions (Grades A/C) was observed (19). This implies that not all changes in PSCR require immediate surgery.

## Clinical implications and future directions

Despite limitations encountered, the findings suggest modern PPV, often combined with other interventions such as laser photocoagulation and tamponade, may be effective for PSCR complications. Most patients undergoing surgery appear to experience some improvement in vision. Given the high-risk complex retinal detachments and PVR, surgeons should counsel patients about the likelihood of multiple interventions. The relative success rate of combined SB/PPV suggests that adjuncts may be considered when traction is extensive (15–18). Importantly, treatment decisions may have to be based on ocular findings rather than haemoglobin type as outcomes appear similar across sickle cell genotypes (15–21). 

Longitudinal cohort data suggest that PSCR may follow a dynamic natural history, with many lesions remaining stable or undergoing spontaneous regression and only a minority progressing to vision-threatening complications (5). Although genotype strongly predicts disease incidence, lesion behaviour over time is heterogenous once PSCR is established (5). Consequently, further development of standardised outcome metrics and staging systems could improve comparability between centres as well as across studies. In particular, refined and universally applied “new sea-fan” grading systems may allow more precise distinction and comparability of neovascular morphology, activity, and progression in PSCR. Earlier peripheral vascular classifications, such as the Penman system, focused primarily on structural border abnormalities and did not fully account for lesion activity or prognostic variability (22). 

Building on these limitations, Sayag et al. introduced a novel sea-fan classification within a prospective clinical trial, distinguishing early, active neovascular lesions from more fibrosed and inactive forms, and demonstrated that lesion stage influenced both progression and response to intervention (19). Future research is warranted to refine management. The field lacks comparisons of medical/non-surgical care or different surgical approaches. Randomised controlled trials (RCTs) would help establish the most efficient strategy for different PSCR stages (18). Further development of outcome metrics and standardised stages, such as “new sea-fan” grades, would facilitate comparisons between centres. Meanwhile, recent data support the need for an initial observation in very early lesions, and prompt vitrectomy if vitreous haemorrhage or traction detachment occurs (19). This suggests that anatomical reattachment may remain an important indicator of potential visual recovery.

## Conclusion

Recent studies suggest that surgical intervention for PSCR may yield favourable anatomical reattachment rates and could improve vision in many eyes. However, studies are limited by their retrospective nature, and outcomes are less predictable in complex detachment cases. It remains important for clinicians to weigh the benefits of surgery against complications such as PVR and cataract and to inform patients accordingly. Although the current data is promising, the need for rigorous trials and prospective studies is required to guide the evidence–based management of PSCR.

## Data Availability

The original contributions presented in the study are included in the article/**Supplementary Material**. Further inquiries can be directed to the corresponding author.
