# Supplementary material for: Surgical management of sickle cell retinopathy: a systematic review of clinical outcomes and a single-arm meta-analysis of single-surgery anatomical success
Source: Front Ophthalmol (Lausanne). 2026 Jul 23;6:1842211. doi: 10.3389/fopht.2026.1842211 (PMC13441773; doi:10.3389/fopht.2026.1842211)

Results

Classical Meta-Analysis

Model Summary

Meta-Analytic Tests

	Test	p
Heterogeneity	$Q_e(3)$ = 2.94	.400
Pooled effect	t(3) = 4.47	.021

Meta-Analytic Estimates

		95% CI		95% PI	
	Estimate	Lower	Upper	Lower	Upper
Pooled effect	1.027	0.295	1.758	0.295	1.758
τ	4.670×10^{-4}	0.000	1.889		
τ^2	2.181×10^{-7}	0.000	3.567		

Forest Plot

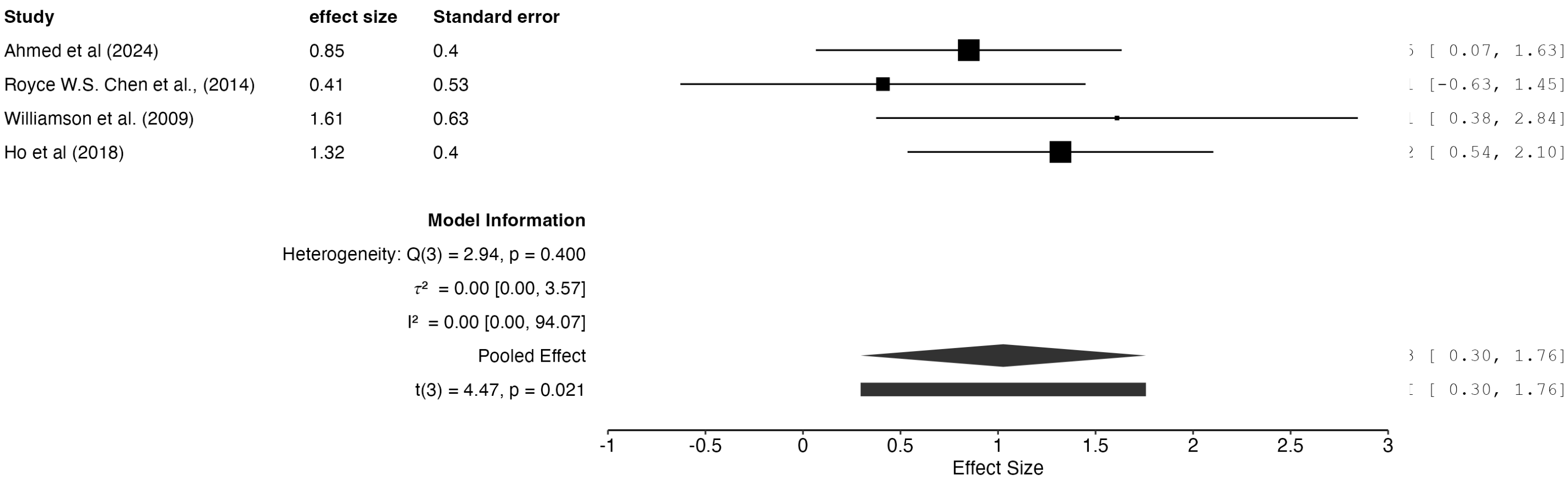

Supplement: Supplementary file 1 [file DataSheet1.pdf]
