# Supplementary material for: Surgical management of sickle cell retinopathy: a systematic review of clinical outcomes and a single-arm meta-analysis of single-surgery anatomical success
Source: Front Ophthalmol (Lausanne). 2026 Jul 23;6:1842211. doi: 10.3389/fopht.2026.1842211 (PMC13441773; doi:10.3389/fopht.2026.1842211)

# Results

## Classical Meta-Analysis

### Model Summary

*Meta-Analytic Tests*

| | Test | p |
|---|---|---|
| Heterogeneity | $Q_e(3)$ = 5.10 | .165 |
| Pooled effect | t(3) = 3.75 | .033 |

*Meta-Analytic Estimates*

| | | 95% CI | | 95% PI | |
|---|---|---|---|---|---|
| | Estimate | Lower | Upper | Lower | Upper |
| Pooled effect | 0.918 | 0.140 | 1.697 | −0.393 | 2.229 |
| $\tau$ | 0.332 | 0.000 | 1.824 | | |
| $\tau^2$ | 0.110 | 0.000 | 3.328 | | |

### Forest Plot

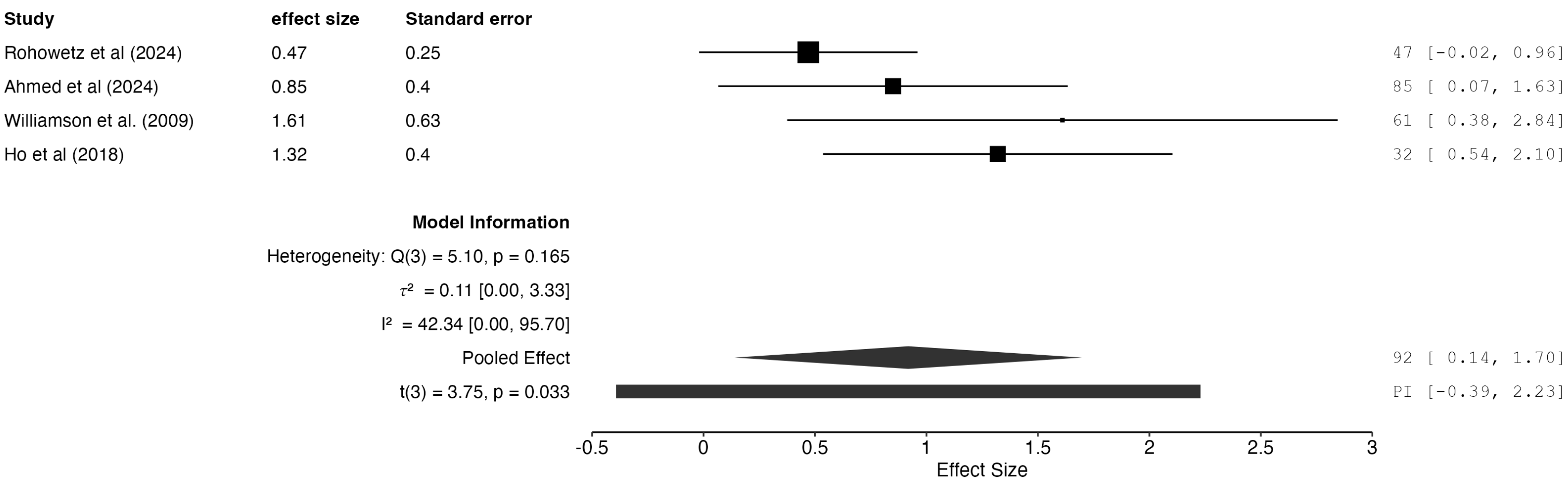

Supplement: Supplementary file 2 [file DataSheet2.pdf]
