# Supplementary material for: Surgical management of sickle cell retinopathy: a systematic review of clinical outcomes and a single-arm meta-analysis of single-surgery anatomical success
Source: Front Ophthalmol (Lausanne). 2026 Jul 23;6:1842211. doi: 10.3389/fopht.2026.1842211 (PMC13441773; doi:10.3389/fopht.2026.1842211)

Results

Classical Meta-Analysis

Model Summary

Meta-Analytic Tests

	Test	p
Heterogeneity	$Q_e(3)$ = 3.30	.348
Pooled effect	t(3) = 3.29	.046

Meta-Analytic Estimates

		95% CI		95% PI	
	Estimate	Lower	Upper	Lower	Upper
Pooled effect	0.648	0.021	1.274	0.021	1.274
τ	0.003	0.000	1.979		
τ^2	6.395×10^{-6}	0.000	3.915		

Forest Plot

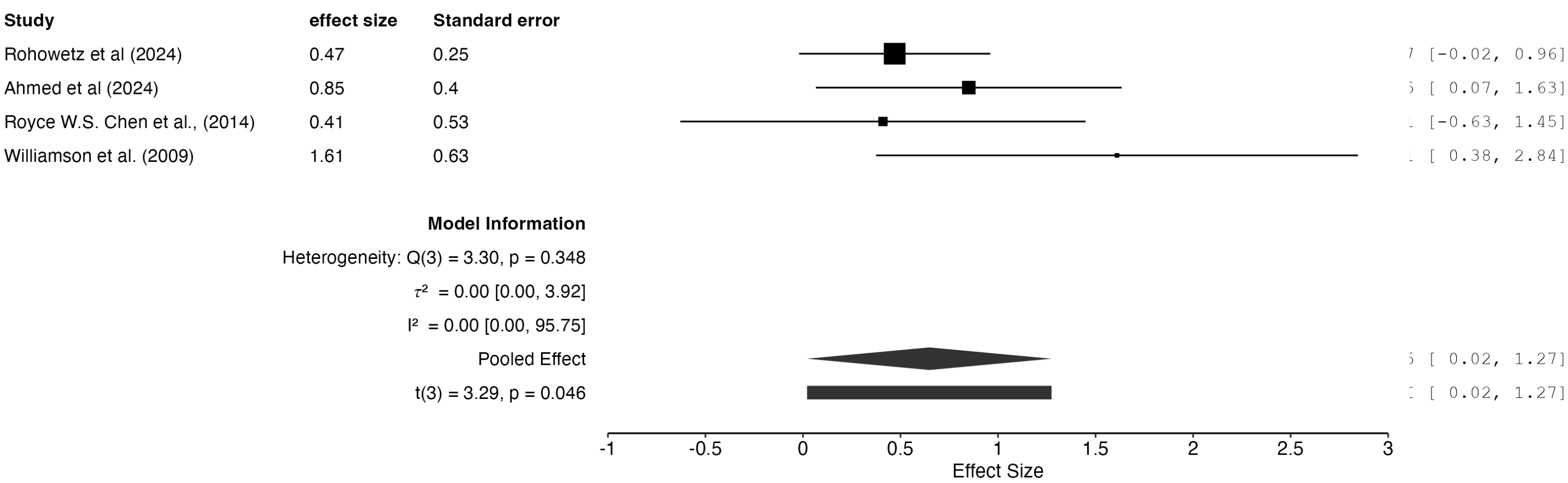

Supplement: Supplementary file 3 [file DataSheet3.pdf]
