# Supplementary material for: Surgical management of sickle cell retinopathy: a systematic review of clinical outcomes and a single-arm meta-analysis of single-surgery anatomical success
Source: Front Ophthalmol (Lausanne). 2026 Jul 23;6:1842211. doi: 10.3389/fopht.2026.1842211 (PMC13441773; doi:10.3389/fopht.2026.1842211)

Results

Classical Meta-Analysis

Model Summary

Meta-Analytic Tests

	Test	p
Heterogeneity	$Q_e(3)$ = 3.69	.297
Pooled effect	t(3) = 3.63	.036

Meta-Analytic Estimates

		95% CI		95% PI	
	Estimate	Lower	Upper	Lower	Upper
Pooled effect	0.732	0.089	1.375	−0.192	1.657
τ	0.209	0.000	1.509		
τ^2	0.044	0.000	2.277		

Forest Plot

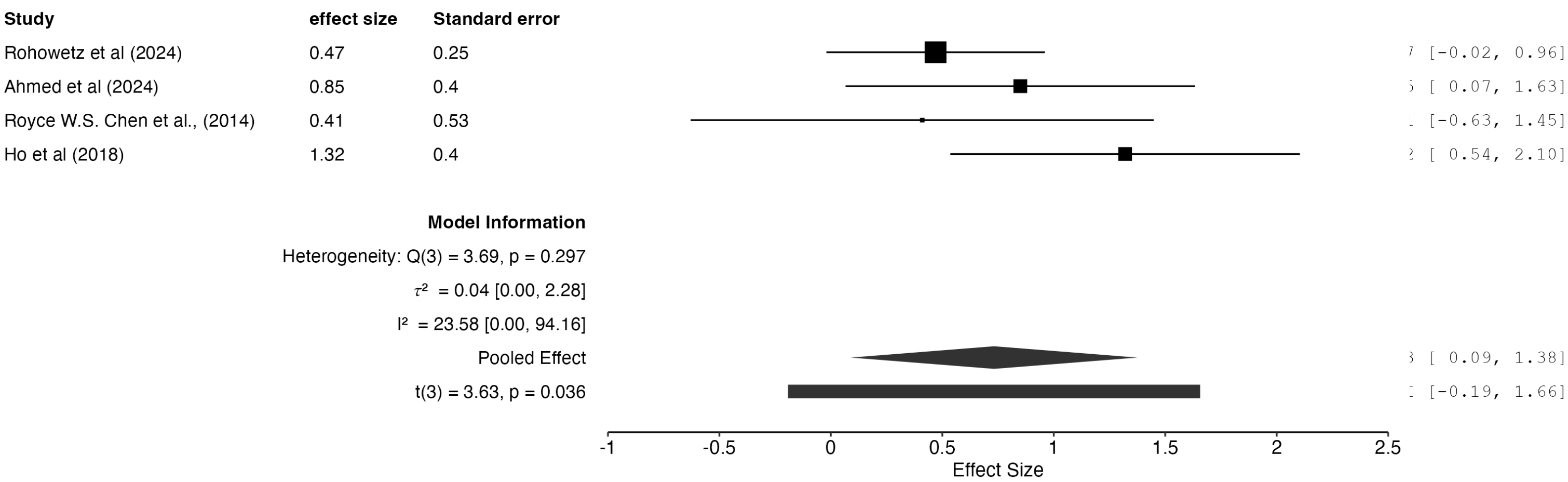

Supplement: Supplementary file 4 [file DataSheet4.pdf]
