# Supplementary material for: Surgical management of sickle cell retinopathy: a systematic review of clinical outcomes and a single-arm meta-analysis of single-surgery anatomical success
Source: Front Ophthalmol (Lausanne). 2026 Jul 23;6:1842211. doi: 10.3389/fopht.2026.1842211 (PMC13441773; doi:10.3389/fopht.2026.1842211)

Results

Classical Meta-Analysis

Model Summary

Meta-Analytic Tests

	Test	p
Heterogeneity	$Q_e(4)$ = 5.61	.230
Pooled effect	t(4) = 3.90	.017

Meta-Analytic Estimates

		95% CI		95% PI	
	Estimate	Lower	Upper	Lower	Upper
Pooled effect	0.829	0.239	1.418	−0.111	1.768
τ	0.264	0.000	1.419		
τ^2	0.069	0.000	2.013		

Forest Plot

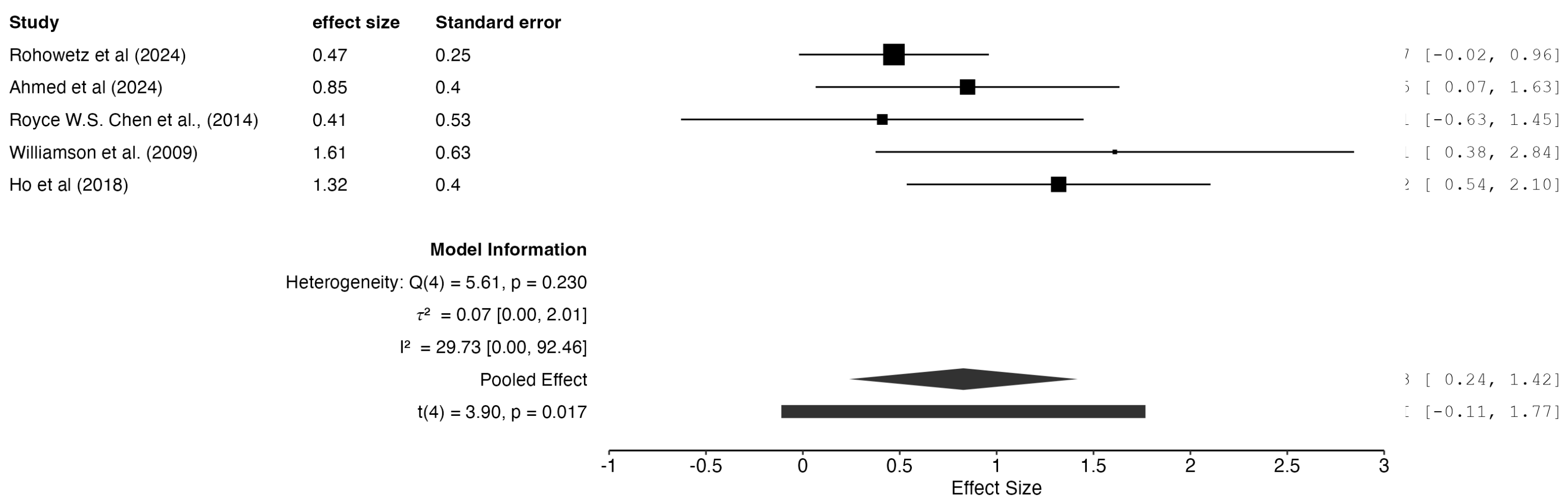

Supplement: Supplementary file 6 [file DataSheet6.pdf]
