# Supplementary material for: Surgical management of sickle cell retinopathy: a systematic review of clinical outcomes and a single-arm meta-analysis of single-surgery anatomical success
Source: Front Ophthalmol (Lausanne). 2026 Jul 23;6:1842211. doi: 10.3389/fopht.2026.1842211 (PMC13441773; doi:10.3389/fopht.2026.1842211)
Supplement: Supplementary file 7 [file Table4.docx]

**Surgical Management of Sickle Cell Retinopathy: A Systematic Review of Clinical Outcomes**

**Search Strategy for three main databases**

**PubMed: 106 records retrieved**
("sickle cell disease"[MeSH Terms] OR "sickle cell anemia"[MeSH Terms] OR "sickle cell"[Title/Abstract])

AND
("retinopathy"[MeSH Terms] OR "retinopathy"[Title/Abstract] OR "sickle cell retinopathy"[Title/Abstract])

AND
("surgery"[MeSH Terms] OR "surgical procedures, operative"[MeSH Terms] OR "vitrectomy"[MeSH Terms]

OR
"photocoagulation"[MeSH Terms] OR "anti-VEGF"[Title/Abstract] OR "laser"[Title/Abstract])

**Embase: 416 records retrieved**
('sickle cell disease'/exp OR 'sickle cell anemia'/exp OR 'sickle cell':ab,ti)

AND
('retinopathy'/exp OR 'sickle cell retinopathy':ab,ti OR 'retinopathy':ab,ti)

AND
('surgery'/exp OR 'vitrectomy'/exp OR 'photocoagulation'/exp OR 'anti VEGF agent'/exp

OR
'anti-VEGF':ab,ti OR 'laser':ab,ti)

**Cochrane CENTRAL: 1 record retrieved**
("sickle cell" AND "retinopathy") AND
(surgery OR surgical OR vitrectomy OR photocoagulation OR laser OR anti-VEGF)

All references will be imported into Rayyan for deduplication and screening. Full-text screening will be done independently by two reviewers. Discrepancies will be resolved through consensus or third-party adjudication (supervisor).
